# Mismatch between the proposed ability concepts of the Graduate Record Examination and the critical thinking skills of physical therapy applicants suggested by an expert panel in the United States

**DOI:** 10.3352/jeehp.2019.16.24

**Published:** 2019-08-27

**Authors:** Emily Shannon Hughes

**Affiliations:** Department of Physical Therapy, College of Health Professions, University of Tennessee Health Science Center, Memphis, TN, USA; Hallym University, Korea

**Keywords:** Physical therapist education, Graduate Records Examination, Critical thinking, Delphi technique, United States

## Abstract

**Purpose:**

The Graduate Record Examination (GRE) is a general examination predictive of success in US-based graduate programs. Used to assess students’ written, mathematical, and critical thinking (CT) skills, the GRE is utilized for admission to approximately 85% of US physical therapist education (PTE) programs. The purpose of this study was to assess whether the CT skills measured by the GRE match those deemed by an expert panel as the most important to assess for PTE program acceptance.

**Methods:**

Using a modified E-Delphi approach, a 3-phase survey was distributed over 8 weeks to a panel consisting of licensed US physical therapists with expertise on CT and PTE program directors. The CT skills isolated by the expert panel, based on Facione’s Delphi Report, were compared to the CT skills assessed by the GRE.

**Results:**

The CT skills supported by the Delphi Report and chosen by the expert panel for assessment prior to acceptance into US PTE programs included clarifying meaning, categorization, and analyzing arguments. Only clarifying meaning matched the CT skills from the GRE.

**Conclusion:**

The GRE is a test for general admission to graduate programs, lacking context related to healthcare or physical therapy. The current study fails to support the GRE as an assessment tool of CT for admission to PTE programs. A context-based admission test evaluating the CT skills identified in this study should be developed for use in the admission process to predict which students will complete US PTE programs and pass the licensure exam.

## Introduction

The Delphi Report defines critical thinking (CT) as the “purposeful, self-regulatory judgment which results in interpretation, analysis, evaluation and inference.” The individual processes of interpretation, analysis, evaluation, and inference are the CT skills used to make a decision [1]. In the United States, the 2015 accreditation standards of the Commission on Accreditation of Physical Therapy Education emphasize CT skills in accredited education programs [2]. To educate healthcare providers effectively, professional academic programs must teach psychomotor skills and techniques utilized in treating those who have medical conditions, as well as the clinical reasoning skills needed to make decisions using CT [3].

The Graduate Record Examination (GRE) General Test, which assesses verbal, qualitative, and analytical factors including CT, is used as an admissions requirement by approximately 85% of physical therapist education (PTE) programs [4]. It is generally used for admission to graduate or business school because it is indicative of success in these types of graduate programs. The aim of this study was to assess whether the Delphi Report’s CT skills that were identified by an expert panel as absolutely essential for assessment before entrance into PTE programs in the United States matched those contained in the GRE, a common PTE admission requirement.

## Methods

### Ethics statement

The research was approved by the Institutional Review Board of University of Tennessee Health Science Center (#16-05037-XP UM). Informed consent was obtained from participants.

### Conceptual framework

With a mixed-methods approach, containing both qualitative and quantitative research methods, the Delphi method used in this study allows a group’s individual opinions, which may change based on feedback, to stand as a solitary opinion. The social group for this study was US licensed physical therapists, and the worldview was their perceptions of CT [5]. The current study was bounded by the Delphi Report’s definitions of skills and sub-skills of CT [1]. These are found in Table 1.

### Setting

**Fig. 1.**
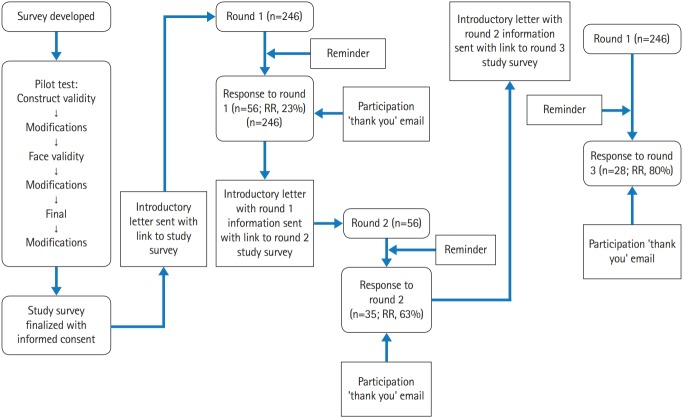
Methods flowchart. RR, response rate percentage.

Since the intent of this study was to find a consensus, the modified E-Delphi method using a Qualtrics survey was the process undertaken for this study [6]. The modified E-Delphi method uses an internet-based survey to replace paper, pencil, and postage [7]. Because no previous study has assessed these variables in PTE, a survey was developed to gather this information. The survey was pilot-tested for construct validity, ensuring that it measured what it was designed to measure [8]. Face validity was established by having a faculty colleague at the University of Tennessee who has published in the realm of CT, but is not a physical therapist, assess the survey. In this capacity, colleagues are able to inform the researcher if “the items look OK to them” [8]. Changes to the initial survey were made based on this feedback such as clarifying instrument instructions so that participants would know to rank each skills individually versus ranking skills against each other and adding a progress bar to the survey. A final pilot test was sent to a group of 16 healthcare faculty members at the local health science university where the primary researcher is employed. Over a 4-week period, the 3 rounds of the modified E-Delphi survey were completed. The last pilot test was used to establish the criterion percentage of agreement that was used in the current study (Fig. 1).

### Instrument

An introductory letter was sent via email to all of the experts, disclosing the intent, significance, and methods of the study as well as operational definitions pertaining to the study. In the introductory email, a secure hyperlink to the online research survey was included. If participants clicked the hyperlink, an informed consent document was the first item they were required to complete. The consent document included the purpose of the research, how the participants were selected, the risk involved, and assurance that the participant could withdraw at any time (Appendix 1). The survey platform Qualtrics (https://www.qualtrics.com/) was used.

### Sample and expert panel

A purposive sample of 246 US physical therapists was invited to participate in this study as the expert panel. The experts in this study were defined as published physical therapists in the realm of CT (n=19) and PTE program directors (n=227). Program directors were selected because they are considered to be experts in PTE who have insider knowledge of what CT skills need to be in place prior to admission. Physical therapists who have published in the realm of CT have demonstrated that they have knowledge and expertise in CT, which increased their likelihood of completing the Delphi process [9]. The following inclusion criteria were used: US-licensed physical therapists who had publications on CT in the last 20 years; otherwise, US-licensed program directors of accredited PTE programs. Opting out of the informed consent or the survey process or failing to complete the survey excluded participants from the study.

Fifty-six physical therapists completed the entire first round of the study, yielding a 23% response rate. The second survey for round 2 was sent to the 56 participants from round 1. Thirty-five participants completed round 2 of the survey, for a response rate of 63%. The final survey was sent to the 35 panel members who completed round 2. With a response rate of 80%, 28 panel members completed the final round of this survey. The classification of participants is outlined in Table 2.

A 3-round process was chosen for this modified E-Delphi survey because the literature suggests that surveys with more than 3 rounds can cause participant fatigue and lower the response rate [9].

In each round, the expert panel provided demographic information and answered survey questions by ranking each of the CT skills using a 5-point Likert scale. The CT skills and definitions were given because research has shown that providing definitions to the experts can significantly reduce the time invested in completing a Delphi survey and can strengthen the Delphi method [10,11]. Each expert was directed to choose the importance of a CT skill that could be assessed by an examination prior to PTE. The scores on the Likert scale were 0=not important, 1=little importance, 2=average importance, 3=very important, and 4=absolutely essential (Appendix 1).

In the first round, a skill was retained if 90% of the panel rated a skill with a score of 2 or higher. A score of 2 represented the point where a skill or construct was seen as having average importance. The second survey was sent out and the expert panel completed a survey similar to the first round. In the second round, 75% of the experts had to choose a score of least 3 (very important) for the skill to be retained for round 3. In the last round, 75% of the experts had to give a skill or construct a score of at least 3 (very important) for this skill or construct to be viewed as significant enough for inclusion on an admission exam for PTE. Pilot-testing guided the percentage thresholds. For the first round, 90% was chosen because higher percentages would result in more skills being eliminated. Feedback from the pilot group in the first round indicated it was difficult not to consider all skills as absolutely essential. In subsequent rounds, 75% was used to retain skills since pilot-testing indicated that this percentage eliminated some, but not all skills.

## Results

Using a modified E-Delphi approach, a 3-phase survey was distributed over 8 weeks. Analysis of the data from round one eliminated 1 CT skill, analyzing arguments, with an expert panel agreement of 89%. The retained CT skills included categorization, decoding significance, clarifying meaning, examining ideals, detecting arguments, analyzing arguments, assessing claims, assessing arguments, querying evidence, conjecturing alternatives, drawing conclusions, stating results, justifying procedures, presenting arguments, self-examination, and self-correction. Stating results earned the highest agreement score of 100% (Supplement 1.).

Round 2 eliminated 8 CT skills (percentage of agreement): decoding significance (63%), examining ideals (74%), detecting arguments (70%), querying the evidence (63%), conjecturing alternatives (46%), drawing conclusions (74%), justifying procedures (74%), and presenting arguments (74%). The CT skills retained in round 2 were: categorization, clarifying meaning, assessing claims, assessing arguments, stating results, self-examination, and self-correction. As in round 1, the CT skill stating results (88%) had the highest consensus rating (Supplement 1).

Categorization (62%) was the single CT skill eliminated in the last round. The CT skills retained in this final survey were clarifying meaning, assessing claims, assessing arguments, stating results, self-examination, and self-correction (Supplement 1). In total, 10 CT skills were eliminated over the 3 rounds of the modified E-Delphi survey. Then, the CT skills isolated by the expert panel were compared to the CT skills assessed by the GRE. Although the GRE’s CT skills show some similarity to the Delphi Report’s CT skills, information on the framework used to derive the GRE CT skills was not evident in the literature. The specific skills retained or eliminated are found in Table 3.

## Discussion

The expert panel selected clarifying meaning, assessing claims, assessing arguments, stating results, self-examination, and self-correction as the most important CT skills an applicant needs when applying to a PTE program. When the results of the expert panel were compared to the GRE skills, only clarifying meaning, a subskill of interpretation, appeared to be assessed by the GRE and was retained by the expert panel. Clarifying meaning corresponds to the statement made by the developers of the GRE that it evaluates test-takers’ ability to articulate “complex ideas clearly and effectively” [4]. Although the CT skills described by a work of Facione [1] show some similarity to the CT skills described on the GRE, further information on the framework used to derive these CT skills was not evident.

Clarifying meaning may have been a common item between the survey conducted in this study and the GRE CT skills because it is an initial step in CT. It is a useful skill for recognition of problems, as it helps paraphrase or clarify a problem. Successful mastery of this skill can allow a student to remove ambiguity or confusion in communications, in relationships, and in general experiences. For the physical therapy student, clarifying meaning is essential to clinical reasoning, as it allows the student to consider difficulties and options, raise questions, and analyze solutions to make decisions concerning a patient’s health and safety [12,13].

The literature reviewed does not support clarifying meaning as important. In a study of Brudvig et al. [14] using another CT assessment, the Health Science Reasoning Test (HSRT), student physical therapists showed significant improvements in CT between entrance into the program and their third year of school. The skills of drawing conclusions, conjecturing alternatives, and querying the evidence fall under inference, and none of these skills were chosen by the expert panel as important to assess prior to entrance into PTE programs. Huhn et al. [3], also using the HSRT, assessed differences in the CT of novice (first-year students) versus expert (at least 5 years of experience and clinical specialization) physical therapists. That study found that examining ideals, detecting arguments, and analyzing arguments were significantly different in the novice and expert groups. All these skills were also eliminated by the expert group in the current study. These works assessed CT changes in established physical therapy students versus students prior to entrance into PTE. Many of the CT skills are facilitated through the actual didactic and clinical context of PTE. No literature was found that studied CT prior to start of PTE. This may explain why clarifying meaning was not found in the literature.

The skills measured on the GRE do not have a healthcare-related focus, and therefore may not show an applicant’s true grasp of CT. Many applicants prepare for PTE programs by majoring in exercise science, kinesiology, or a science, technology, engineering, and math program and by observing or working in a physical therapy clinic. Taking an examination, void of the healthcare context in which they have submerged themselves for years, could put these applicants at a disadvantage.

In conclusion, based on the comparisons made in this study, the GRE does not include most of the skills that experts felt were needed. The context for the GRE is not specific to healthcare or physical therapy. Due to these variables and the inconsistent use in US PTE programs, the CT of applicants cannot truly be assessed by this examination. Currently, there is no pre-admission examination specific to physical therapy or one that has CT as part of its focus. However, studies have suggested that assessing CT during the admission process would be beneficial for predicting which students would be successful in PTE [15,16]. Alternative tests that could be used to assess CT include the HSRT [17], the California Critical Thinking Skills Test [17], and the Watson Glaser Critical Thinking Test [18], of which only the HSRT has a healthcare focus. Nonetheless, a partnership formed by physical therapist educators, examination developers, and physical therapist experts in CT, clinical reasoning, and clinical decision-making could use the results of this study to form the basis for the CT portion of a discipline-specific admission examination for PTE.

## Figures and Tables

**Table 1.** Critical thinking skills and sub-skills as defined by the Delphi Report [11]

Critical thinking skills	Critical thinking skills defined	Subskills	Subskills defined
Interpretation	The ability to understand and convey the significance of an experience	Categorization	Occurs when experiences or beliefs are framed for understanding
		Decoding significance	A situation or experience is described in relation to affective attitudes or motives behind the situation
		Clarifying meaning	Restating or paraphrasing the situation or experience in different terms to remove any ambiguity or confusion
Analysis	Concepts or situations are examined, and relationships are identified	Examining ideals	Ideals are compared and contrasted, and problems with the ideals are identified and broken down
		Detecting arguments	Determining whether an idea or situation involves reasons to support or refute the idea
		Analyzing arguments	A complex process where the conclusion, the reasons for the conclusion, support for those reasons and their structure, other outcomes, and outliers are identified and accepted or rejected
Evaluation	Deciding whether a person or their statements are credible or finding that relationships are logical	Assessing claims	Recognizing factors that make the source of information credible
		Assessing arguments	Judging whether an argument is plausible or false
Self-regulation	The metacognitive activity of assessing one’s analysis, judgements, and evaluation	Self-examination	Looking at the reasoning used, and opinions created, as well as the “motivation, values, attitudes and interests” that determine the outcome
		Self-correction	Occurs when self-examination shows an error in the decision or reason, and allows for correction of this mistake
Explanation	The results of reasoning are stated and justified based on the evidence examined to reach a decision	Stating results	Giving accurate statements
		Justifying procedures	Presenting the evidence behind the decision
		Presenting arguments	Giving a rationale for accepting an assertion
Inference	Components are assembled for a hypothesis, then considered, and a conclusion is made	Querying evidence	Occurs when additional supporting information is needed to develop or reinforce an argument and how to find that additional supporting information
		Conjecturing alternatives	Creating other alternative ways to ask a question, multiple ways to resolve an issue or project consequences
		Drawing conclusions	Ensues when hypotheses are tested or opinions are compared to determine what to do or believe

**Table 2.** Response rate and classification of participants

Variable	Round 1	Round 2	Round 3
No. of participants	246	56	35
Responded (response rate %)	56 (23)	35 (63)	28 (80)
Gender (female/male)	40/16	27/8	21/17
% PD/faculty/other^a)^	80/11/9	77/11/12	78/11/11
Published CT^b)^ (%)	20 (36)	12 (34)	9 (32)

PD, program directors.

a) Admissions committee or PD designee.

b) Critical thinking, self-identified.

**Table 3.** Retained or eliminated skills

Skills	Round 1 (n=56)	Round 2 (n=35)	Round 3 (n=28)	Retained
Categorization	95	77	62	x
Decoding significance	95	63	x	x
Clarifying meaning	95	86	76	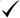
Examining ideals	98	74	x	x
Detecting arguments	98	70	x	x
Analyzing arguments	89	x	x	x
Assessing claims	98	83	79	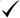
Assessing arguments	96	77	79	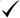
Querying the evidence	95	63	x	x
Conjecturing alternatives	95	46	x	x
Drawing conclusions	98	74	x	x
Stating results	100	88	93	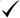
Justifying procedures	98	74	x	x
Presenting arguments	93	74	x	x
Self-examination	98	77	90	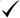
Self-correction	95	86	79	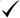

Values are presented as %. Percentages are agreement rates of the expert panel. X areas signify eliminated skills.

## Supplementary materials

Supplement 1. Data files are available from https://doi.org/10.7910/DVN/VRWOJF

Supplement 2. Audio recording of the abstract.
