# Supplementary material for: Mismatch between the proposed ability concepts of the Graduate Record Examination and the critical thinking skills of physical therapy applicants suggested by an expert panel in the United States
Source: J Educ Eval Health Prof. 2019 Aug 27;16:24. doi: 10.3352/jeehp.2019.16.24 (PMC6760996; doi:10.3352/jeehp.2019.16.24)
Supplement: Supplementary file 2 [file jeehp-16-24-app.pdf]

Appendix 1. Delphi survey questionnaire

Q1. Consent to Participate in a Research Study [redacted]

- **WHY ARE YOU BEING INVITED TO TAKE PART IN THIS RESEARCH?** You are being invited to take part in a research study about critical thinking and higher order constructs in the admission process to physical therapist education programs.

- **WHO IS DOING THE STUDY?** The person in charge of this study is Shannon Hughes, a doctoral student at the [redacted]. She is being guided in this research by [redacted].

- **WHAT IS THE PURPOSE OF THIS STUDY?** By doing this study, we hope to learn which critical and higher order constructs should be adopted into a pre-admission exam, if such an examination is adopted for use for admission into physical therapist education programs.

- **ARE THERE REASONS WHY YOU SHOULD NOT TAKE PART IN THIS STUDY?** None

- **WHERE IS THE STUDY GOING TO TAKE PLACE AND HOW LONG WILL IT LAST?** The research procedures will be conducted at [redacted] through an online Qualtrics survey. The study should last approximately two to three months.

- **WHAT WILL YOU BE ASKED TO DO?** This is the first of three surveys, where you will be asked to rank the critical thinking skill and higher order construct that you feel is the most essential to be tested prior to admission to physical therapist education programs. The internet questionnaire should take approximately ten to twenty minutes to complete. Other questions included in this initial survey will ask demographic information including gender, age, educational information, number of years as a physical therapist and number of years of interest in critical thinking and higher order constructs.

- **WHAT ARE THE POSSIBLE RISKS AND DISCOMFORTS?** None

- **WILL YOU BENEFIT FROM TAKING PART IN THIS STUDY?** You will not get any personal benefit from taking part in this study.

- **DO YOU HAVE TO TAKE PART IN THE STUDY?** If you decide to take part in the study, it should be because you really want to volunteer. You will not lose any benefits or rights you would normally have if you choose not to volunteer. You can stop at any time during the study and still keep the benefits and rights you had before volunteering.

- **IF YOU DON'T WANT TO TAKE PART IN THE STUDY, ARE THERE OTHER CHOICES?** If you do not want to be in the study, there are no other choices except not to take part in the study.

- **WHAT WILL IT COST YOU TO PARTICIPATE?** There are no costs associated with taking part in the study.

- **WILL YOU RECEIVE ANY REWARDS FOR TAKING PART IN THIS STUDY?** You will not receive any rewards or payment for taking part in the study.

- **WHO WILL SEE THE INFORMATION THAT YOU GIVE?** We will make every effort to keep private all research records that identify you to the extent allowed by law. Your information will be combined with information from other people taking part in the study. When we write about the study, we will write about the combined information we have gathered. You will not be personally identified in these written materials. We will publish the results of this study; however, we will keep your name and other identifying information private. This study is anonymous. That means that no one, other than the researcher, will know that the information you give came from you.

- **CAN YOUR TAKING PART IN THE STUDY END EARLY?** If you decide to take part in the study you still have the right to decide at any time that you no longer want to continue. You will not be treated differently if you decide to stop taking part in the study. The individuals conducting the study may need to withdraw you from the study. This may occur if you are not able to follow the directions they give you, if they find that your being in the study is more risk than benefit to you, or if the agency funding the study decides to stop the study early for a variety of scientific reasons.

- **WHAT IF YOU HAVE QUESTIONS, SUGGESTIONS, CONCERNS, OR COMPLAINTS?** Before you decide whether to accept this invitation to take part in the study, please ask any questions that might come to mind now. If you have concerns or questions about this study, please contact Shannon Hughes at [redacted]. If you have any questions about your rights as a volunteer in this research, contact the [redacted]

- By beginning this survey, you acknowledge that you have read this information and agree to participate in this research, with the knowledge that you are free to withdraw your participation at any time without penalty.

❍ Yes, I AGREE to participate in this study. (1)
❍ No, I DO NOT agree to participate in this study. (2)

If No, I DO NOT agree to parti... Is Selected, Then Skip To End of Survey

Q5. How many years have you been a physical therapist?

❍ 0–1 years (1) ❍ 1–5 years (2) ❍ 6–10 years (3) ❍ 11–15 years (4)
❍ 16–20 years (5) ❍ 21–25 years (6) ❍ 26–30 years (7) ❍ > 30 years (8)

Q2. Gender?

❍ Male (1) ❍ Female (2) ❍ Choose not to answer (3)

Q3. What is your age?

❍ Under 18 (1) ❍ 18–24 (2) ❍ 25–34 (3) ❍ 35–44 (4)
❍ 45–54 (5) ❍ 55–64 (6) ❍ 65–74 (7) ❍ 75–84 (8)
❍ 85 or older (9)

Q4. What is the highest degree or level of education you have completed?

❍ High school graduate (1) ❍ Completed some college (2) ❍ Associate degree (3)
❍ Bachelor's degree (4) ❍ Completed some postgraduate (5) ❍ Master's degree (6)
❍ Ph.D., law or medical degree (7)

Q9. What is your entry level physical therapy degree?
❍ Certificate (1) ❍ Bachelor's degree (2) ❍ Entry- level Master's degree (3) ❍ Entry-level Doctoral degree (4)

Q6. Are you a physical therapist education program director?
❍ Yes (1) ❍ No (2)

Answer If Are you a physical therapist education program director? Yes Is Selected And If you are not a program director, please indicate your role. Faculty member Is Selected

Q8. How many years have you participated in physical therapy education?
❍ 0–1 years (1) ❍ 1–5 years (2) ❍ 6–10 years (3) ❍ 11–15 years (4)
❍ 16–20 years (5) ❍ 21–25 years (6) ❍ 26–30 years (7) ❍ > 30 years (8)

Q7. How many years have you been interested in critical thinking and higher order thinking/constructs?
❍ 0–1 years (1) ❍ 1–5 years (2) ❍ 6–10 years (3) ❍ 11–15 years (4)
❍ 16–20 years (5) ❍ 21–25 years (6) ❍ 26–30 years (7) ❍ > 30 years (8)

Answer If Are you a physical therapist education program director? No Is Selected

Q10. If you are not a program director, please indicate your role.
❍ Clinical practice (1) ❍ Faculty member (2)
❍ Other (3) ____________________

Q13. In the next sections, you will be presented with the critical thinking skills and the sub skills defined in Facione's (1990), The Delphi Report. Please read the definition of the critical thinking skill, and in your opinion which skill is the most important to have PRIOR to entrance to a physical therapy program. Please keep in mind, these are the skills that you feel a student should possess BEFORE beginning physical therapy school.

Q11. Interpretation is the ability to understand and convey the significance of an experience. The skills that make up interpretation are:
- Categorization: occurs when experiences or beliefs are framed for better understanding decoding significance: the situation or experience is described in relation to affective attitudes or the motive behind situation
- Clarifying meaning: restating or paraphrasing the situation or experience in different terms to remove any ambiguity or confusion

Q14. Categorization: occurs when experiences or beliefs are framed for better understanding
❍ Not important (1) ❍ Little importance (2) ❍ Average importance (3)
❍ Very important (4) ❍ Absolutely essential (5)

Q15. Decoding significance: the situation or experience is described in relation to affective attitudes or the motive behind situation
❍ Not important (1) ❍ Little importance (2) ❍ Average importance (3)
❍ Very important (4) ❍ Absolutely essential (5)

Q16. Clarifying meaning: restating or paraphrasing the situation or experience in different terms to remove any ambiguity or confusion

❍ Not important (1) ❍ Little importance (2) ❍ Average importance (3)
❍ Very important (4) ❍ Absolutely essential (5)

Q17. Analysis is when concepts or situations are examined, and relationships are identified. The skills that make up analysis are: examining ideals: when ideas are compared and contrasted, and problems with the idea are identified and broken down detecting arguments: determining if an idea or situation has reasons to support or refute it analyzing arguments: a complex process where the conclusion, the reasons for the conclusion, support for those reasons and their structure, other outcomes, and outliers are identified and accepted or rejected

Q18. Examining ideals: when ideas are compared and contrasted, and problems with the idea are identified and broken down

❍ Not important (1) ❍ Little importance (2) ❍ Average importance (3)
❍ Very important (4) ❍ Absolutely essential (5)

Q19. Detecting arguments: determining if an idea or situation has reasons to support or refute it

❍ Not important (1) ❍ Little importance (2) ❍ Average importance (3)
❍ Very important (4) ❍ Absolutely essential (5)

Q20. Analyzing arguments: a complex process where the conclusion, the reasons for the conclusion, support for those reasons and their structure, other outcomes, and outliers are identified and accepted or rejected

❍ Not important (1) ❍ Little importance (2) ❍ Average importance (3)
❍ Very important (4) ❍ Absolutely essential (5)

Q21. Evaluation is assessing the credibility of perceptions and logic of the relationships by assessing claims or arguments. The skills that make up evaluation are:

- Assessing claims: recognizing factors that make the source of information credible
- Assessing arguments: judging if an argument is plausible or false

Q24. Assessing claims: recognizing factors that make the source of information credible

❍ Not important (1) ❍ Little importance (2) ❍ Average importance (3)
❍ Very important (4) ❍ Absolutely essential (5)

Q23. Assessing arguments: judging if an argument is plausible or false

❍ Not important (1) ❍ Little importance (2) ❍ Average importance (3)
❍ Very important (4) ❍ Absolutely essential (5)

Q25. Inference uses querying evidence, finding alternatives and drawing conclusions to identify what is needed to make conclusions, or form hypotheses. Skills that make up inference are:

- Querying evidence: occurs when additional support information is needed to develop or reinforce an argument and how to find that additional support information
- Conjecturing alternatives: creating other alternative ways to ask a question, multiple ways resolve an issue or project consequences
- Drawing conclusions: ensues when hypothesis are tested or opinions are compared to determine what to do or believe

Q26. Querying evidence: occurs when additional support information is needed to develop or reinforce an argument and how to find that additional support information

- ❍ Not important (1) ❍ Little importance (2) ❍ Average importance (3)
- ❍ Very important (4) ❍ Absolutely essential (5)

Q27. Conjecturing alternatives: creating other alternative ways to ask a question, multiple ways resolve an issue or project consequences

- ❍ Not important (1) ❍ Little importance (2) ❍ Average importance (3)
- ❍ Very important (4) ❍ Absolutely essential (5)

Q28. Drawing conclusions: ensues when hypothesis are tested or opinions are compared to determine what to do or believe

- ❍ Not important (1) ❍ Little importance (2) ❍ Average importance (3)
- ❍ Very important (4) ❍ Absolutely essential (5)

Q44. An explanation is to declare or justify reasoning by stating the results, justifying the procedures and presenting arguments based on the context. The skills that make up explanation are:

- Stating results: giving accurate statements
- Justifying procedures: presenting the evidence behind a decision
- Presenting arguments: giving reasons to accept a claim or decision

Q29. Stating results: giving accurate statements

- ❍ Not important (1) ❍ Little importance (2) ❍ Average importance (3)
- ❍ Very important (4) ❍ Absolutely essential (5)

Q30. Justifying procedures: presenting the evidence behind a decision

- ❍ Not important (1) ❍ Little importance (2) ❍ Average importance (3)
- ❍ Very important (4) ❍ Absolutely essential (5)

Q32. Presenting arguments: giving reasons to accept a claim or decision

- ❍ Not important (1) ❍ Little importance (2) ❍ Average importance (3)
- ❍ Very important (4) ❍ Absolutely essential (5)

Q45. Self-regulation applies the "skills in analysis and evaluation" (p. 10) to monitor one's own cognitive activities through self-examination and self-correction. The skills that make up self-regulation are:

- Self-examination: looking at the reasoning used, and opinions created, as well as "motivation, values, attitudes and interests" that determine the outcome
- Self-correction: occurs when self-examination shows an error in the decision or reason, and allows for correction of this mistake

Q31. Self-examination: looking at the reasoning used, and opinions created, as well as "motivation, values, attitudes and interests" that determine the outcome

- ❍ Not important (1) ❍ Little importance (2) ❍ Average importance (3)
- ❍ Very important (4) ❍ Absolutely essential (5)

Q33. Self-correction: occurs when self-examination shows an error in the decision or reason, and allows for correction of this mistake

- ❍ Not important (1) ❍ Little importance (2) ❍ Average importance (3)
- ❍ Very important (4) ❍ Absolutely essential (5)
